# Development and Characterization of Polymorphic Genic-SSR Markers in *Larix kaempferi*

**DOI:** 10.3390/molecules20046060

**Published:** 2015-04-08

**Authors:** Xing-Bin Chen, Yun-Hui Xie, Xiao-Mei Sun

**Affiliations:** 1 State Key Laboratory of Tree Genetics and Breeding, Chinese Academy of Forestry, Xiangshan Rd., Beijing 100091, China; 2 Research Institute of Forestry, Chinese Academy of Forestry, Xiangshan Rd., Beijing 100091, China; E-Mails: xingbinsd@163.com (X.-B.C.); xieyunhui33@163.com (Y.-H.X.)

**Keywords:** *Larix kaempferi*, unigene sequences, simple sequence repeat (SSR), polymorphism, cross-species amplification

## Abstract

New simple sequence repeat (SSR) markers were developed in the Japanese larch (*Larix kaempferi*) using unigene sequences for further genetic diversity studies and the genetic improvement of breeding programs. One thousand two handred and thirty five (1235) primer pairs were tested and 165 successfully identified in *L. kaempferi*. Out of the amplified candidate markers, 145 (90.6%) exhibited polymorphism among 24 individuals of *L. kaempferi*, with the number of alleles per locus (N_a_), observed heterozygosity (H_o_), expected heterozygosity (H_e_) and polymorphic information content (PIC) averaging at 4.510, 0.487, 0.518 and 0.459, respectively. Cross-species amplification of randomly selection of 30 genic-SSRs among the 145 polymorphic ones showed that 80.0% of the SSRs could be amplified in *Larix olgensis*, 86.7% could be amplified in *Larix principi-rupprechtii* and 83.0% could be amplified in *Larix gmelinii*. High rates of cross-species amplification were observed. The genic-SSRs developed herein would be a valuable resource for genetic analysis of *Larix kaempferi* and related species, and also have the potential to facilitate the genetic improvement and breeding of larch.

## 1. Introduction

*Larix kaempferi*, which is one of the most valuable conifers in boreal and temperate forests, is originally native to Japan and was introduced in artificial plantations in China at the end of the 19th century as a successful exotic species [1,2]. It is of great ecological and economical importance, being highly appreciated for the properties of its wood, including high mechanical strength, an attractive reddish color and high natural durability [3,4]. Due to lack of efficient molecular markers, little is known however about the population genetic diversity and the genetic relationships among larch germplasm and breeding populations.

Microsatellites, or simple sequence repeat (SSR) markers, are abundantly dispersed within the genome and have been widely used as genetic markers. SSR markers are very useful for a spectrum of genetic and breeding applications because of their reproducibility, co-dominant inheritance, multi-allelic nature and good genome coverage [5,6].

SSR markers can be isolated not only from the intergenic regions but also from the transcribed regions of all higher organisms [7]; the latter SSRs are commonly identified as EST-SSRs or genic SSRs [8]. However, the development of genomic SSRs is time comsuming and labour intensive. In recent years, the availability of large sets of expressed sequence tags (ESTs) has given rise to an expedient approach for identification of SSRs, namely, EST-SSRs. SSRs can be directly sourced from such databases, thereby reducing time and cost for microsatellite development. Besides such advantages of SSR markers, EST-SSRs represent transcribed regions of the genome and are likely to be conserved and transferable across taxa [9], thus EST-SSRs development in one species can be used in related species for marker development. A major disadvantage of the EST-derived microsatellites is the sequence redundancy that yields multiple sets of markers at the same locus. This problem can be circumvented by assembling the ESTs into a unique gene sequences called unigenes [10]. The unigene-based microsatellite markers, or genic-SSRs, would therefore, have unique identity in the transcribed regions of the genome and which can be used for accurately assaying functional diversity in the natural populations and the available germplasm collections as well as for comparative mapping and evolutionary studies as anchor markers [11].

In the case of *L. kaempferi*, three chloroplast SSRs [12], 19 genomic SSRs [2] and six EST-SSRs [13] have been developed, respectively. Recently, Li *et al*. have designed suitable PCR primers for 3595 SSRs coming from 146,786 *L. kaempferi* transcripts [14]. Microsatellite marker-based genetic studies, such as genetic maps construction, quantitative trait loci (QTL) mapping and marker-assisted selection in *L. kaempferi* are generally restricted due to limited availability of these markers.

Here, we make use of the large dataset of unigene sequences to identify a large number of genic-SSRs for *L. kaempferi.* The specific aims of our study were to: (i) characterize the genic-SSR loci in *L. kaempferi* and evaluate SSR primers and polymorphisms in different wild-type individuals; (ii) test cross-species transferability within *Larix* and develop functional markers that could be used for germplasm identification, genome mapping, and gene tagging for economic traits in larch species.

## 2. Results and Discussion

**Figure 1:**
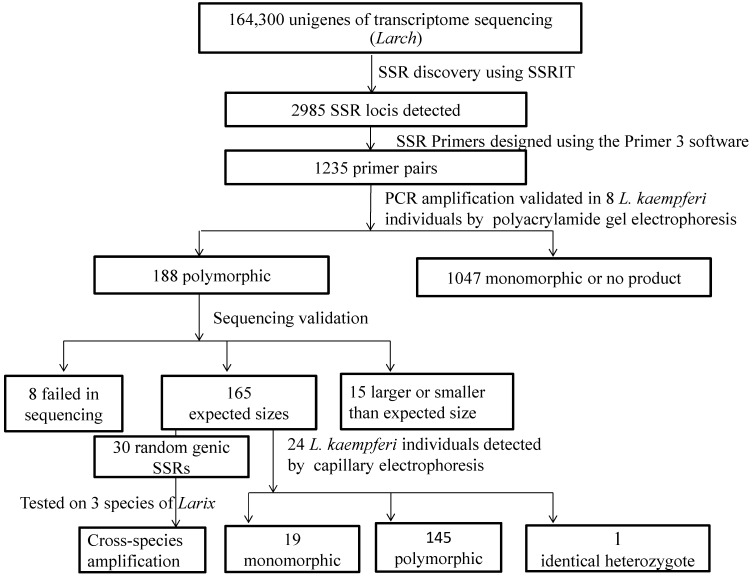
Flow diagram of *L. kaempferi* genic-SSRs development and applications in this study.

A total of 2985 putative SSRs were identified in 2703 of 164,300 unigenes using SSRIR software, with an average frequency of one SSR per 55.04 unigenes. Genic-SSRs with trinucleotides were the most abundant type (815; 27.3%), followed by hexanucleotides (524; 17.6%) and dinucleotides (490; 16.4%). In total, 1036 SSR motifs were detected (data not shown), in which AT/TA, AG/TC, and AGC/TCG ranked the most frequent, accounting for 7.0%, 5.9%, 3.0% of the total of putative EST-SSRs, respectively. After excluding the fragments with too short or inappropriate flanking sequences, 1235 putative SSRs were successfully designed from SSR flanking regions using Primer 3 program (Figure 1). Of the 1235 genic-SSRs identical in sequence to the original ESTs, none of the primers have been described previously.

Using eight individuals of *L. kaempferi*, 188 (15.2%) of 1235 primer pairs were identified that yielded stable, clear, repeatable and polymorphic amplicons in 6% polyacrylamide gels. The other primer pairs were monomorphic or gave no product, and so were excluded from further analysis (Figure 1). Those with single clear fragments from a individual were directly sequenced. Finally, 180 amplicons were successfully sequenced with genic-SSR primer pairs, whereas the remaining eight failed in sequencing due to too weak amplification. Sequence alignment revealed that 165 amplified PCR products of the expected sizes, with sequence identity 80.3%–100.0% and presence of the expected repeat motifs (Table S1), however eight and seven PCR products were larger or smaller, respectively, than expected and absenced the expected SSRs (Figure 1). Analysis of all 165 genic-SSR loci revealed that hexanucleotide (44, 26.6%), dinucleotide (37, 22.4%) and trinucleotide repeat motifs (34, 20.6%) predominated (Table S1). The GenBank accession numbers of 165 genic-SSR loci were also provided in Table S1. Moreover, in terms of SSR position, 99 (60.0%), 28 (17.0%) and 27 (16.3%) were found in coding sequence (CDS), 5' untranslated rigion (5'UTR) and 3'UTR, respectively (Table S2). Furthermore, BlastX searches against the NCBI nonredundant protein database under the default settings showing that 24 (14.6%) of the 165 genic-SSRs were homologous to known genes and 22 (13.3%) corresponded to predicted or hypothetical proteins while 119 (72.1%) produced no significant match (*E* ≤ 10^−5^; Table S2).

In capillary electrophoresis, 19 primers amplified monomorphic products (one band), one marker had identical heterozygotes (two bands), and 145 primers generated clean and reproducible polymorphic bands among all 24 individuals (Figure 1 and Table S2). The 20 genic-SSRs showed polymorphism in polyacrylamide gel electrophoresis, but they did not show polymorphism in capillary electrophoresis, since the eight trees used in polyacrylamide gel electrophoresis were different from the 24 individuals genotyped by capillary electrophoresis. A total of 676 unique alleles were identified (Table S2). Polymorphism estimation indicated that number of alleles per locus (N_a_), observed heterozygosity (H_o_), expected heterozygosity (H_e_) and polymorphic information content (PIC) were 2–13 (mean 4.510), 0.0–1.0 (mean 0.487), 0.042–0.903 (mean 0.518) and 0.040–0.874 (mean 0.459), respectively (Table S2). The polymorphism estimates are over six EST-SSRs for genus *Larix* [13], but less than those of *L. kaempferi* for 19 SSRs [2]. We obtained 59 (40.7%) highly polymorphic markers according to the criterion of PIC ≥ 0.5 as defined [15]. Forty-seven loci deviated significantly from Hardy Weinberg Equilibtrum (*p* < 0.001), indicating the presence of population structure and absence of panmixia in these 24 individuals detected.

All tested SSR primer pairs displayed a high amplification frequency across *larix species.* Of the 30 examined SSR markers, 21 (70.0%) successfully amplified in all species. The transferability of the 30 primers tested in *Larch* species were 80.0% (*L. olgensis*), 86.7% (*L. principis–rupprechtii*) and 83.0% (*L. gmelinii*) (Table S3)*.* Thus, the developed genic-SSR markers could be applied within *L. kaempferi* and provided data on polymorphisms among related species. High rates of cross-species amplification of genic-SSR markers have been demonstrated in several studies, where 39 of the 45 (86.67%) genic-SSR primers developed in radish showed transferability to one or more of the nine related *Brassica* species tested [16], and where 93 of 108 SSR primers (86.1%) adopted from other *Prunus* species were transferable to chokecherry [17]. In generally, a greater cross-taxon rate of genic-based markers is expected due to the conservative nature of transcribed regions [18,19].

## 3. Experimental Section 

### 3.1. Plant Materials and DNA Extraction

Thirty two (32) individuals of *L. kaempferi* (28 from a parent population is made up of 163 *L. kaempferi* individuals, the four others from a progeny population produced by open pollination of the parent population), were randomly selceted for genetic polymorphism and three related species in *Larix* (*Larix olgensis*, *Larix principis-rupprechtii*, *larix gmelini*, two in each species) for transferability studies. All of the materials were sampled from the Da GuJia National Larch Breeding Centre, Liaoning Province, China (124°47′–125°12′E, 42°22′–42°16′N). Genomic DNA from 32 individuals of *L. kaempferi*, and three related species of *Larix* was extracted from leaves of each individual, using a modified CTAB method [13].

### 3.2. Novel Genic-SSR Identification and Primer Design

One hundred and sixty four thousand three hundred (164,300) unigenes retrieved from the transcriptome sequencing of larch [20,21] were used for SSR exploitation by SSRIR software [22] with the following criteria: at least 6, 5, 5, 4, 3 and 2 SSR motif repeat units for di-, tri-, tetra-, pentra-, hexa-, hepta- and higher-order nucleotides, respectively. Fifty seven thousand five hundred and sixteen (57,516) unigenes are the result of 572,403 high-quality reads’ twice Assemblies using the Contig Assembly Program, CAP3 [23]. The 572,403 reads were generated from a 454 sequencing cDNA library of *L. kaempferi* somatic embryo [20]. The remaining 106,784 unigenes are result of 910,607 high-quality reads assembly using Newbler software (provided with the Roche GS FLX sequencer). These 910,607 high-quality reads were generated from the four cDNA libraries that were constructed from two clones at two important stages of adventitious root development of *L. kaempferi × L. olgenisis* [21]. After excluding the fragments with too short or inappropriate flanking sequences, Primer pairs flanking the SSRs were designed using Primer 3 program [24] with a length of 18–25 bp, amplification product size of 100–500 bp, annealing temperature (T) ranged from 54 °C to 60 °C, and GC content between 40% and 60%.

### 3.3. PCR Amplification and Functional Annotations

PCR amplifications were carried out in a Veriti thermal cycler (Applied Biosystems, Foster City, CA, USA) in a total volume of 15 µL, which contained 20 ng DNA, 1*×* PCR buffer, 2 mM MgCl_2_, 10 mMdNTPs, 10 pmol each primer, and 0.5 Unit Taq DNA polymerase (TaKaRa, Dalian, China). The thermal program was as follows: 4 min at 94 °C, then 30 cycles of 45 s at 94 °C, 45 s at 56 °C and 45 s at 72 °C, finishing with 7 min at 72 °C. The fragments resulting from the PCR amplifications of eight individuals of *L. kaempferi* (four from the parent poplation, the other four from the progeny population) were electrophoretically separated in 6% polyacrylamide gels and then were visualized by silver staining following the protocols [25].

Polymorphic amplicons proved by polyacrylamide gels were separated in agarose gel electrophoresis, and then directly sequenced using Big Dye Terminator 3.1 (BDT3.1) and an ABI 3730xl sequencer (Applied Biosystems). We have performed the DNAMAN 5.2.2 (Lynnon Biosoft, Point-Claire, QC, Canada) to implement Sequence alignment. And then using the online server ORF Finder [26]. determined SSR position. Furthermore, genic-SSRs associated unigene sequences were blasted against the NCBI nonredundant protein database using BLASTX [27] with an expected value (E-value) of 10^−5^ for the function of polymorphic genic-SSRs.

### 3.4. Polymorphism Detection

For further verifying the accuracy of the polymorphisms of novel genic-SSRs, the forward primer was labeled with one fluorescent dye and tested on 24 unrelated *L. kaempferi* individuals that came from the parent poplation. And then we preformed capillary electrophoresis using an ABI3730xl DNA Analyzer with GeneScan-500LIZ size standard (Applied Biosystems). Based on the data result, alleles sizes were determined using GeneMaker software (SoftGenetics, State College, PA, USA). Number of alleles per locus (N_a_), observed heterozygosity (H_o_), expected heterozygosity (H_e_) and polymorphic information content (PIC) were calculated with the MSA software [28]. Hardy-Weinberg equilibrium (HWE) was calculated with Genepop 4.2 [29].

### 3.5. Detection of the Transferability of Genic-SSR Primers

To assess the utility of this SSR marker set beyond *L. kaempferi*, 30 polymorphic primer pairs were amplified from three related species of *Larix* using the above PCR conditions. The fragments resulting from the PCR amplifications were detected by 6% polyacrylamide gel electrophoresis.

## 4. Conclusions 

A total of 165 *L. kaempferi* genic-SSRs were developed and sequence-verified, and 145 proved high polymorphic in *L. kaempferi.* High cross-species amplifications were characterized for a set of the genic-SSR loci. Thus, these markers will be useful for germplasm characterization, genome mapping, and gene tagging for economic traits in *L. kaempferi* and may have potential for genetics and breeding applications in *L. kaempferi* and related species.

## Supplementary Materials

Supplementary materials can be accessed at: http://www.mdpi.com/1420-3049/20/04/6060/s1.

## References

[1] Pâques L.E., García-Casas M.C., Charpentier J.-P. (2013). Distribution of heartwood extractives in hybrid larches and in their related European AND Japanese larch parents: Relationship with wood colour parameters. Eur. J. For. Res..

[2] Isoda K., Watanabe A. (2006). Isolation and characterization of microsatellite loci from *Larix kaempferi*. Mol. Ecol. Notes.

[3] Lai M., Sun X., Chen D., Xie Y., Zhang S. (2014). Age-related trends in genetic parameters for *Larix kaempferi* and their implications for early selection. BMC Genet..

[4] Sun X.M., Zhang S.G., Zhou D.Y., Wang X.D., Ding B., Liu S.M. (2008). Phenological variation of larix species and their intra-species and inter-species hybrid families and early selection. Scientia Silvae Sinicae.

[5] Powell W., Machray G.C., Provan J. (1996). Polymorphism revealed by simple sequence repeats. Trends Plant Sci..

[6] Silva P.I., Martins A.M., Gouvea E.G., Pessoa-Filho M., Ferreira M.E. (2013). Development and validation of microsatellite markers forBrachiaria ruziziensisobtained by partial enome assembly of Illumina single-end reads. BMC Genomics.

[7] Sraphet S., Boonchanawiwa A., Thanyasiriwat T., Boonseng O., Tabata S., Sasamoto S., Shirasawa K., Isobe S., Lightfoot D.A., Tangphatsornruang S. (2011). SSR and EST–SSR based genetic linkage map of cassava (Manihot esculentaCrantz). Theor. Appl. Genet..

[8] Li Y.C., Korol A.B., Fahima T., Nevo E. (2004). Microsatellites within genes: Structure, function, and evolution. Mol. Biol. Evol..

[9] Varshney R.K., Graner A., Sorrells M.E. (2005). Genic microsatellite markers in plants: Features and applications. Trends Biotechnol..

[10] Parida S.K., Anand R., Kumar K., Dalal V., Singh N.K., Mohapatra T. (2006). Unigene derived microsatellite markers for the cereal genomes. Theor. Appl. Genet..

[11] Korir N.K., Han J., Shangguan L.F., Wang C., Kayesh E., Zhang Y.Y., Fang J.G. (2013). Plant variety and cultivar identification: Advances and prospects. Crit. Rev. Biotechnol..

[12] Zhang X., Shiraishi S., Huang M. (2004). Analysis of Genetic Structure in Population of *Larix kaempferi* by Chloroplast SSR Markers. Hereditas.

[13] Yang X., Sun X., Zhang S. (2011). Short note: Development of Six EST-SSR Markers in Larch. Silvae Genet..

[14] Li W.F., Han S.Y., Qi L.W., Zhang S.G. (2014). Transcriptome resources and genome-wide marker development for Japanese Larch (*L. kaempferi*). Front. Agric. Sci. Eng..

[15] Botstein D., White R.L., Skolnick M., Davis R.W. (1980). Construction of a genetic linkage map in man using restriction fragment length polymorphisms. Am. J. Hum. Genet..

[16] Zhai L.L., Xu L., Wang Y., Cheng H., Chen Y.L., Gong Y.Q., Liu L.W. (2014). Novel and useful genic-SSR markers from de novo transcriptome sequencing of radish (*Raphanus sativus* L.). Mol. Breed..

[17] Wang H.X., Walla J.A., Zhong S.B., Huang D.Q., Dai W.H. (2012). Development and cross-species genera transferability of microsatellite markers discovered using 454 genome sequencing in chokecherry (*Prunus virginiana* L.). Plant Cell Rep..

[18] Ellis J., Burke J. (2007). EST-SSRs as a resource for population genetic analyses. Heredity.

[19] Wu J., Cai C.F., Cheng F.Y., Cui H.L., Zhou H. (2014). Characterisation and development of EST-SSR markers in tree peony using transcriptome sequences. Mol. Breed..

[20] Zhang Y., Zhang S., Han S., Li X., Qi L. (2012). Transcriptome profiling and in silico analysis of somatic embryos in Japanese Larch (*Larix leptolepis*). Plant Cell Rep..

[21] Han H., Sun X., Xie Y., Feng J., Zhang S. (2014). Transcriptome and proteome profiling of adventitious root development in hybrid larch (*Larix kaempferi × Larix olgenisis*). BMC Plant Biol..

[22] Temnykh S., de Clerck G., Lukashova A., Lipovich L., Cartinhour S., McCouch S. (2001). Computational and experimental analysis of microsatellites in rice (*Oryza sativa* L.): Frequency, length variation, transposon associations, and genetic marker potential. Genome Res..

[23] Huang X., Madan A. (1999). CAP3, A DNA sequence assembly program. Genome Res..

[24] Rozen S., Skaletsky H., Misener S., Krawetz S. (2000). Primer3 on the WWW for general users and for biologist programmers. Methods in Molecular Biology, Vol. 132: Bioinformatics Methods and Protocols.

[25] Charters Y., Wilkinson M. (2000). The use of self-pollinated progenies as ‘in-groups’ for the genetic characterization of cocoa germplasm. Theor. Appl. Genet..

[26] ORF Finder (Open Reading Frame Finder). http://www.ncbi.nlm.nih.gov/gorf/gorf.html.

[27] BLAST. http://www.ncbi.nlm.nih.gov/BLAST.

[28] Dieringer D., Schlötterer C. (2003). Microsatellite analyzer (MSA): A platform independent analysis tool for large microsatellite data sets. Mol. Ecol. Notes.

[29] Rousset F. (2008). GENEPOP’007: A complete re-implementation of the GENEPOP software for Windows and Liuux. Mol. Ecol. Resour..

