# Supplementary material for: Development and Characterization of Polymorphic Genic-SSR Markers in Larix kaempferi
Source: Molecules. 2015 Apr 8;20(4):6060–7. doi: 10.3390/molecules20046060 (PMC6272221; doi:10.3390/molecules20046060)
Supplement: Supplementary file 1 [file molecules-20-06060-s001.pdf]

Supplementary Materials

Table S1. The sequence of forward and reverse primers, Mg^{2+} concentration, annealing temperature (Tm), repeat motif and sequence identity of the 165 genic-SSRs developed in *Larix kaempferi*.

Serial No.	Genic-SSR Locus	NCBI Accession	Primer Sequence (5'-3')	Mg^{2+} (mM)	Tm (°C)	Target Size (bp)	Repeat Motif in EST	Target Size (Length Resequenced)(bp)	Identity (%)
1	LARKeSSRH002	KP863070	AGGAGGCGGTTCAGTTCAG<br>GACCTCCTGGGATTTGGATT	1.5	56	132	$(AGC)_6$	141	80.3
2	LARKeSSRH008	KP863071	GAGATGTACACAGTCCGCCC<br>CCTGTTCGGATCCACAGAAT	1.5	56	377	$(ACTGGGC)_4$	417	99
3	LARKeSSRH028	KP863072	TGCCCATTTGAATCCTTAACA<br>TCGTTGTAGAAGAATGGGGC	1.5	56	200	$(AAAATGTGAC)_2$	210	99
4	LARKeSSRH029	KP863073	TGGAGTTGCACACTACGAGG<br>GTGATCGGGAGTTCATCGAC	1.5	56	261	$(AAAGGACCTC)_2$	270	89
5	LARKeSSRH034	KP863074	AACACACCTGGCCCTGTAAG<br>GCGCTGTATTGTATTGATAAGGC	1.5	56	106	$(AAACTCTTC)_3$	126	93
6	LARKeSSRH042	KP863075	GGACACTTTTCTGCTTCCCA<br>CAGGTGGCAGAGTACCCACT	1.5	56	333	$(AAATAG)_3$	343	92
7	LARKeSSRH045	KP863076	CGCCACCTTCCCTATTTACA<br>CCCCAACCCTAAGACACAGA	1.5	56	270	$(AAATATATAT)_2$	281	99
8	LARKeSSRH046	KP863077	ATGTTTTTGGGTTTTGGAGC<br>CAGGTTTATAGCTTTGGTTTGGA	1.5	56	168	$(AAAATTCTTT)_2$	174	95
9	LARKeSSRH052	KP863078	AGGGATGGTTGCTGTGGTAG<br>CATTTCTCCGAGTGGGTTGT	1.5	56	341	$(AATG)_6$	353	98
10	LARKeSSRH057	KP863079	GGACGTCTTAAGCATGCCA<br>AAAGTTCGAAGTGAAGCGGA	1.5	56	119	$(AT)_{11}$	134	85
11	LARKeSSRH094	KP863080	CTGATGGCACATAGCTGCAC<br>CTTGACAAAGGAGCCAAAGC	1.5	56	263	$(ACATAGTAGG)_2$	274	95
12	LARKeSSRH106	KP863081	AGCAGCTGTTGTTGTTGTGG<br>TGCAAATCGTCTTCACAAGC	1.5	56	251	$(AGCAT)_4$	249	98
13	LARKeSSRH122	KP863082	TGCTTCCGCAGATATAGCCT<br>CTAAGTTTGTGCGCCGAGAT	1.5	56	242	$(ACCCTC)_6$	253	98

Table S1. *Cont.*

Serial No.	Genic-SSR Locus	NCBI Accession	Primer Sequence (5'-3')	Mg^{2+} (mM)	Tm (°C)	Target Size (bp)	Repeat Motif in EST	Target Size (Length Resequenced)(bp)	Identity (%)
14	LARKeSSRH125	KP863083	TCTCCCAACCACCCAAGTTA<br>TCAGGTTCTGGGTTTGGTTC	1.5	56	243	$(AT)_{11}$	257	80
15	LARKeSSRH128	KP863084	TGGCCAATTTTGAGTTCAAGT<br>AGAGGTCTCGTAACGGCAGA	1.5	56	247	$(AAATTGGCCT)_{2}$	258	99
16	LARKeSSRH131	KP863085	GAAGATCACAACAAAGGGCG<br>TGTCCAGGCAACTGAAACAG	1.5	56	291	$(AGATG)_{5}$	258	96
17	LARKeSSRH136	KP863086	GGGACGTACTGAGACCGTGT<br>TCATTAACTGGGCATGTGGA	1.5	56	364	$(AACCAACCAG)_{2}$	376	95
18	LARKeSSRH137	KP863087	ATACATATTCCTTCCGGCCC<br>TTGGAAAGACTCCAGGATGG	1.5	56	178	$(AAATAAGC)_{2}$	183	93
19	LARKeSSRH140	KP863088	GGAGTAGTGCATATGGGCGT<br>TATGCTTTTTCCCAGCCAAC	1.5	56	294	$(AAAGCC)_{3}$	325	80
20	LARKeSSRH147	KP863089	AAATGAAGAACCCGAACACG<br>AGCTCTCGATTCATGGCTGT	1.5	56	191	$(AGC)_{8}$	205	97
21	LARKeSSRH149	KP863090	CAAGGAGAACTGAAGGCTGG<br>TTTCTCGTCAACTGAGGGCT	1.5	56	256	$(ACGGCACTCC)_{2}$	273	90
22	LARKeSSRH168	KP863091	ACTTCAGTATCACCCGCCAC<br>CGATCTTTCGGCTCTTATCG	1.5	56	159	$(AGCAGG)_{5}$	172	80.5
23	LARKeSSRH177	KP863092	TGGCTTTTGCAACAAGTGAC<br>GGCCATCCTCTGTCATGATT	1.5	56	407	$(AAATAGCTTC)_{2}$	415	98
24	LARKeSSRH179	KP863093	AACACCAAAGTTGCTGGGAC<br>GGCTGAGGATTATGATCGGA	1.5	56	334	$(AAAGAAGTTC)_{2}$	354	93
25	LARKeSSRH180	KP863094	ACATCCTCCCCTTGGTCTCT<br>CTTGCTCCTGGCGAAGTAAC	1.5	56	177	$(AAAGATACC)_{2}$	196	98
26	LARKeSSRH182	KP863095	CTGATCAGGGTGAGATGGGT<br>GCTGCTGTTGTTGTTGCTGT	1.5	56	319	$(AAACCC)_{3}$	326	93
27	LARKeSSRH187	KP863096	AGATTTGGAAGCAGCAGGAA<br>AAGTTGTTCAGCCCATCTCG	1.5	56	145	$(AACAGC)_{5}$	156	96

Table S1. *Cont.*

Serial No.	Genic-SSR Locus	NCBI Accession	Primer Sequence (5'-3')	Mg^{2+} (mM)	Tm (°C)	Target Size (bp)	Repeat Motif in EST	Target Size (Length Resequenced)(bp)	Identity (%)
28	LARKeSSRH189	KP863097	GTAAGGAGGAGGATTGCGGT<br>AGTTCATCCTTCTGGCTGGA	1.5	56	266	$(ACTGGC)_6$	274	97
29	LARKeSSRH191	KP863098	TTGAATTCGTCCTGGGTCTC<br>GTCTGAACGACGAAGAAGGC	1.5	56	154	$(AACCCTCCC)_2$	166	97
30	LARKeSSRH197	KP863099	TTAGCAAAAGTCTTCGCCGT<br>ACGAAACTACGCGGATGAAC	1.5	56	339	$(AAACGGACGG)_2$	301	96
31	LARKeSSRH206	KP863100	TGCAGTTCGTGTTGCTAACC<br>CCACCTGGCGAAGTATTGAT	1.5	56	362	$(AACAATAATT)_2$	342	97
32	LAREeSSRH217	KP863101	ATCCCAAGAACCGATATCCC<br>TGACCGATTTTCTCTCGCTT	1.5	56	432	$(ACGCCC)_3$	437	97
33	LARKeSSRH221	KP863102	AGATTCGGTTTTCATGGACG<br>GCAAGCGAGAGAAAGCAGTT	1.5	56	387	$(AGCATC)_3$	406	97
34	LARKeSSRH224	KP863103	GCTGCCCAGGTGAAGAATAC<br>TCCCAATTTCACAATCATAGGAG	1.5	56	183	$(AACGTCC)_3$	188	95
35	LARKeSSRH233	KP863104	AGGGGCAGGCTTAATCACTT<br>GATTCGAAGAAAATTGCCCA	1.5	56	456	$(ATCCCC)_4$	467	94
36	LARKeSSRH236	KP863105	GAATGCCATTGGAACAGCTT<br>TGCCTGTGCTCGTTCATAAG	1.5	56	303	$(AGC)_8$	316	85
37	LARKeSSRH239	KP863106	AATAGTTTGGGGAACCGACC<br>CCCTGGTTCTATTGACGCAT	1.5	56	338	$(AATCCAGTG)_2$	340	84
38	LARKeSSRH251	KP863107	GTTGTTCAGCCCATTTCGAT<br>AGATTTGGAAGCAGCAGGAA	1.5	56	125	$(AACAGC)_3$	151	90
39	LARKeSSRH253	KP863108	AACGGGGTTATCAAGCACTG<br>ATGCGTTTCATTCGATCCTC	1.5	56	364	$(AGGATC)_3$	374	99
40	LARKeSSRH256	KP863109	TATCCGGCACCCCTGTAATA<br>GGTTTGATGGGAAACTGCAT	1.5	56	121	$(AGCCCC)_4$	130	97
41	LARKeSSRH264	KP863110	CCGACGCTATTCCCAACTAA<br>CTTGGAAGGCTATGGCTACG	1.5	56	105	$(AGATGG)_3$	125	100

Table S1. *Cont.*

Serial No.	Genic-SSR Locus	NCBI Accession	Primer Sequence (5'-3')	Mg^{2+} (mM)	Tm (°C)	Target Size (bp)	Repeat Motif in EST	Target Size (Length Resequenced)(bp)	Identity (%)
42	LARKeSSRH274	KP863111	CGGACGAATAGATCCCAGAA<br>ATGAGGCAGGGTCGTGTTAG	1.5	56	249	$(AGCCC)_5$	270	84
43	LARKeSSRH276	KP863112	GAACCAAACCCAGAACCTGA<br>CTGGGGATATAAATGGGGCT	1.5	56	186	$(AACCGG)_3$	190	100
44	LARKeSSRH279	KP863113	AATTCAGGGGACATTGCTTG<br>TTTCTGGGTCTCAGGAATGG	1.5	56	179	$(AATCGATGC)_2$	195	92
45	LARKeSSRH283	KP863114	TCTAGCCATGTGCATTGTCC<br>ATTCTGTGTTTTGTCGCACG	1.5	56	340	$(AAAGATGAC)_2$	350	98
46	LARKeSSRH299	KP863115	CGATCTTTCGGCTCTTATCG<br>ACTTCAGTATCACCCGCCAC	1.5	56	171	$(AAGGAG)_3$	172	85
47	LARKeSSRH301	KP863116	CCAAGGAAACCAGTGCATTT<br>CATTGGTTGAGGTGGAGGAG	1.5	56	267	$(AATGGC)_4$	270	99
48	LARKeSSRH309	KP863117	AATGGGCTCTCAATGCAATC<br>AGGTGACAAATGGGACCAAG	1.5	56	470	$(ACCTCC)_3$	474	98
49	LARKeSSRH339	KP863118	AATTCGTTGGCCTTCAGATG<br>CGATCTCGGGCATTATGAGT	1.5	56	318	$(AGC)_7$	332	99
50	LAREeSSRHL003	KP863119	TGTGGTCATTGGTGGACATT<br>GAGTCCCACATTTGCAGGTT	1.5	56	324	$(AAGAT)_4$	332	85
51	LAREeSSRHL004	KP863120	AGATGAGCTCCTGTTGGGAA<br>TTGCTTTGCAGCTTACCAGA	1.5	56	222	$(AACCTC)_7$	227	100
52	LAREeSSRHL006	KP863121	TGCGTTCTGTGTGTCTCTCC<br>GGGTAGGCCTGAAGAAGGCT	1.5	56	105	$(AAT)_{10}$	90	90
53	LAREeSSRHL007	KP863122	GGACGAGACCAATCCAAACT<br>CAAAAGCCGGGAGAAATGTA	1.5	56	236	$(ACAGC)_5$	254	90
54	LAREeSSRHL009	KP863123	GGTCTTAGTCACAGCCCAGC<br>TTCGATCCTTCTGAATTGGC	1.5	56	162	$(AGGATG)_4$	198	86
55	LAREeSSRHL021	KP863124	GGTCACATGGGAATGAGCTT<br>TGACTTGTTATTTCTGAATTTTGGA	1.5	56	169	$(AACAGTCTAG)_2$	183	87

Table S1. *Cont.*

Serial No.	Genic-SSR Locus	NCBI Accession	Primer Sequence (5'-3')	Mg^{2+} (mM)	Tm (°C)	Target Size (bp)	Repeat Motif in EST	Target Size (Length Resequenced)(bp)	Identity (%)
56	LAREeSSRHL034	KP863125	CCTTCCGTTGCAATCTTCAT<br>CTTTCCACACTGCCAAACCT	1.5	56	112	$(AAG)_7$	112	100
57	LAREeSSRHL042	KP863126	GAATCTGAGAGCTCCGGGTA<br>ATCCATGTTTTTGCCTCGAC	1.5	56	118	$(ACGTCC)_3$	124	88
58	LAREeSSRHL046	KP863127	ATCCAACTGGATCCATCAGC<br>CCGGATAAAGTCCAGCAAGA	1.5	56	379	$(AAGCTGTGTC)_2$	388	98
59	LAREeSSRHL062	KP863128	CGGATCTCCTCCTGAATGAA<br>GTTGAGCTGTCGGATCACAA	1.5	56	232	$(AATGCATACT)_2$	245	99
60	LAREeSSRHL079	KP863129	GATTCGAAGAAAATTGCCCA<br>TACCCGTTTCCATTTCCATC	1.5	56	196	$(ATCCCC)_3$	177	96
61	LAREeSSRHL083	KP863130	CCAAACCTCAACAACAGCAA<br>GTGCTGCGGATGAGTACAGA	1.5	56	154	$(AAAATCAAG)_2$	154	99
62	LAREeSSRHL085	KP863131	TTTGGCAGTTTTGACAGTCG<br>CGAGCCATTTGTGTCTTTGA	1.5	56	138	$(AACATTG)_2$	137	90
63	LAREeSSRHL101	KP863132	ATCAAGATCGCCGGTGTTAC<br>GATTGCCAAAGCCAATGC	1.5	56	240	$(AGGCGG)_4$	212	98
64	LAREeSSRHL104	KP863133	CGGATACGGCAAATTTTCAA<br>CCTTTGTCTTGGTGCTGGAT	1.5	56	295	$(ATC)_8$	320	90
65	LAREeSSRHL114	KP863134	AGGAGGCGGTTCAGTTCAG<br>CAACGCCAGATTAGGAGAGC	1.5	56	215	$(AGC)_7$	233	90
66	LAREeSSRHL120	KP863135	GAAAAGGGTGGAAATGCAAA<br>GGCACTACCTAACCAAAGTAGGA	1.5	56	146	$(AAAG)_8$	157	87
67	LAREeSSRHL129	KP863136	ATCTTCCCCTGCTGTTTGTG<br>GGGAGCGTTGAATGGATAGA	1.5	56	254	$(AAAAGCATC)_2$	262	98
68	LAREeSSRHL137	KP863137	GAGGATTGTGCACACCTTGA<br>ATGGGTTTGACAGCGGATAA	1.5	56	109	$(AGC)_7$	139	96
69	LAREeSSRHL138	KP863138	AAGGAGTGGGTTTATTGGGG<br>AGGTGATGATGATGATGTACAATG	1.5	56	249	$(AATCATCAT)_3$	258	91

Table S1. *Cont.*

Serial No.	Genic-SSR Locus	NCBI Accession	Primer Sequence (5'-3')	Mg^{2+} (mM)	Tm (°C)	Target Size (bp)	Repeat Motif in EST	Target Size (Length Resequenced)(bp)	Identity (%)
70	LAREeSSRHL159	KP863139	CACAGACCTCATGACGATGG<br>TTCTGATTCTGCCTCTGGCT	1.5	56	233	$(AGAGCC)_5$	243	86
71	LAREeSSRHL161	KP863140	CGTTTCCAAAATGCCTCAGT<br>ACACCCAGGGAAGCTCCTAT	1.5	56	264	$(AGATGG)_5$	308	87
72	LAREeSSRHL162	KP863141	GGGTCACGTCTACGAGGTTT<br>GCTAGGACTGCCACTGGATT	1.5	56	101	$(AC)_{10}$	126	86
73	LAREeSSRHL163	KP863142	AATGGAAGCGTGAGGACATC<br>TGGTTAAGGGCAACCAAAAG	1.5	56	275	$(AAGGCC)_3$	295	96
74	LAREeSSRHL165	KP863143	TATCCTCCTGCACCATCCTC<br>TCCTCAGTTGCCCTTTGTTT	1.5	56	387	$(AAAGGATGAT)_2$	398	90
75	LAREeSSRHL166	KP863144	CTCAAGAGGTATCAAGCGGC<br>TAAGGGCTAAGTGGGTGCTC	1.5	56	207	$(AAACCCT)_3$	216	97
76	LAREeSSRHL215	KP863145	TAAATACGCACAAGCCCACA<br>GGAGGAGCAAATGGATCAAA	1.5	56	202	$(AGGCGG)_3$	226	81
77	LAREeSSRHL217	KP863146	AATCCAACAGAAGGCCAAGA<br>GCCGGCAAATAGGTTGATATT	1.5	56	211	$(ACTCATATG)_2$	220	86
78	LAREeSSRHL246	KP863147	TGGATGGTAAGAACGCACAG<br>ACTTTTACCCGTTGTGGTCG	1.5	56	177	$(AAT)_9$	192	89
79	LAREeSSRHL272	KP863148	GCAACAACATCGAACAGCAA<br>TGTTTATAGGCCAAGCCACC	1.5	56	354	$(AATATATAT)_2$	371	95
80	LAREeSSRHL275	KP863149	CTACCTAAGTCGGCCCACAA<br>TATCCTCGGAAACCATGAGG	1.5	56	360	$(AAACAAAT)_2$	365	97
81	LAREeSSRHL283	KP863150	CCATTCCCAAACTAAAACGC<br>GATGATGAGGCCCTTCAAAA	1.5	56	199	$(AATGGCAGAC)_2$	235	88
82	LAREeSSRHL299	KP863151	GAACAGCATACAATGGGGCT<br>CGTCGCGAACAAGAATGATA	1.5	56	447	$(AAACCCTACG)_2$	471	98
83	LAREeSSRHL308	KP863152	TGCATTGTCTTGCTGCCTAT<br>ATTGCACTGAATGCACAAGC	1.5	56	285	$(AAACATTT)_2$	320	98

Table S1. *Cont.*

Serial No.	Genic-SSR Locus	NCBI Accession	Primer Sequence (5'-3')	Mg^{2+} (mM)	Tm (°C)	Target Size (bp)	Repeat Motif in EST	Target Size (Length Resequenced)(bp)	Identity (%)
84	LAREeSSRHL346	KP863153	TAGAAAGGGCAAAGGCACTG	1.5	56	109	$(ACCAGC)_4$	124	87
			GGTGCATTTCTCTCCACTCC						
85	LAREeSSRHL357	KP863154	AGGTCCAGCCATTGATGAAG	1.5	56	163	$(AACAGC)_3$	178	95
			TCAATGCAATCCTGGGGTAT						
86	LAREeSSRHL358	KP863155	CTCCCACCTTACCACGAAAG	1.5	56	146	$(AATAATTCTC)_2$	155	93
			TGTGTTAGCATTCCGTGCTC						
87	LAREeSSRHL361	KP863156	GTATGCTGCCAAAGGTGGTT	1.5	56	309	$(ACTC)_5$	321	90
			CATTTCCGGGCTTGTATTTG						
88	LAREeSSRHL366	KP863157	TCGTATCTGGATCTCGGGTT	1.5	56	247	$(ACGGAT)_3$	258	89
			AAAGAGGCAAGCGGTACTCA						
89	LAREeSSRHL372	KP863158	GATTCGGAAATGCGGAAATA	1.5	56	131	$(AAACCCG)_3$	143	90
			AGTTCAAAAATTGGGCGTTG						
90	LAREeSSRHL374	KP863159	AGTTGAACCAACCCTCATCG	1.5	56	312	$(AC)_4$	275	91
			CTGTGGGGTGGAGATCCTTA						
91	LAREeSSRHL380	KP863160	GGCTGGTACATTTCAGGCAT	1.5	56	188	$(AACGGC)_3$	200	97
			AGCCTCTCCTCCTCCTCAAC						
92	LAREeSSRHL391	KP863161	AGCGTATGAATTGGTCCAGG	1.5	56	222	$(ACTGGC)_4$	232	99
			ACGAAGATAGCTCGAACGGA						
93	LAREeSSRHL392	KP863162	GCGGTCAGGCTTTATCTCAG	1.5	56	308	$(AAACAAACAG)_2$	306	95
			ACCTGATGACCACGGGATAG						
94	LAREeSSRHL393	KP863163	GCCAGAACCACCGTTAAAAG	1.5	56	300	$(CCG)_8$	318	94
			AGAGGCGATTATGGGAGCTT						
95	LAREeSSRHL394	KP863164	GGGGAGGTGTTTGACAGAGA	1.5	56	262	$(AAAGGC)_4$	270	99
			AATCAACCGTTGGGAATGAG						
96	LAREeSSRHL395	KP863165	TTTGCTTTAAGCTGGGCAGT	1.5	56	295	$(ACCAGG)_5$	295	87
			CAAAGCTTTCCGAAGGGAAT						
97	LAREeSSRHL396	KP863166	CTTTTGCCCTTTTCCTTTCC	1.5	56	307	$(AAGAGC)_5$	329	95
			TTGTGGGTGTCGTTCACAAT						

Table S1. *Cont.*

Serial No.	Genic-SSR Locus	NCBI Accession	Primer Sequence (5'-3')	Mg^{2+} (mM)	Tm (°C)	Target Size (bp)	Repeat Motif in EST	Target Size (Length Resequenced)(bp)	Identity (%)
98	LAREeSSRHL397	KP863167	CAATGATCGAACTGTGGTTCA<br>GCTCATCTTCAACTTCATGTGG	1.5	56	288	$(AT)_{14}$	290	88
99	LAREeSSRHL398	KP863168	AGTCGGGGATGAAATCTGTG<br>TGTTTCTTTGCGCATACACC	1.5	56	300	$(AGCCTG)_4$	313	98
100	LAREeSSRHL399	KP863169	CTTTGTGTTGCGGGATTCTC<br>TTCCTTTTCCCCTTGGTCTT	1.5	56	299	$(AAAATC)_5$	300	87
101	LAREeSSRHL400	KP863170	GAGGACCTCCTGGCTTTGAT<br>TTAGAGCTGTGTTGCGCTGT	1.5	56	291	$(AGGCGG)_5$	340	99
102	LAREeSSRHL401	KP863171	AGCAGAATAACGAGCCGAAG<br>CCCGCCACTACTCTGCTTAG	1.5	56	302	$(ACCGCC)_3$	314	97
103	LAREeSSRHL402	KP863172	CACATATCTGTGTGTGCCTGTG<br>TTAGGTTGCCAAAACTGCAA	1.5	56	264	$(AC)_{13}$	272	89
104	LAREeSSRHL403	KP863173	TCCATATTGCATAACGCTCCT<br>GCTCCTTCATGTTGTAAGCAAA	1.5	56	297	$(AT)_{11}$	317	90
105	LARKeSSRHL404	KP863174	TCTTGTGACATTCGCCTCTG<br>TCGATGGTGATCTTCACCTG	1.5	56	304	$(AAGCCC)_4$	311	97
106	LAREeSSRQ001	JR170819	GCAAACTCATGTAGACTCGCC<br>CATTGGTGGAACATTGCTTG	1.5	56	184	$(CA)_{10}$	200	90
107	LAREeSSRQ005	JR171181	TTCCCTATTCTCATCCACGG<br>GTCGCCAGTAAATGGCCTTA	1.5	56	250	$(GA)_8$	252	88
108	LAREeSSRQ006	JR171219	CCAAGAAGACCAAAACATCAGA<br>TCTGTCCCTGTTCACAACCA	1.5	56	156	$(AT)_6$	183	90
109	LAREeSSRQ010	JR171974	CCCAGAATGCAATACGGACT<br>TTCCCAAGGAAAATCTGGTG	1.5	56	219	$(TC)_7$	230	97
110	LAREeSSRQ017	JR173000	CCACCTCAAATCTTCTCCCA<br>CCTGCATATGAGTCTGCTGC	1.5	56	139	$(CAG)_5$	140	80
111	LAREeSSRQ020	JR173379	TGATCCGCTTAAGGTAACCAA<br>TTGTGAGTGTTTGTGTCGCA	1.5	56	217	$(AG)_6$	215	90

Table S1. *Cont.*

Serial No.	Genic-SSR Locus	NCBI Accession	Primer Sequence (5'-3')	Mg^{2+} (mM)	Tm (°C)	Target Size (bp)	Repeat Motif in EST	Target Size (Length Resequenced)(bp)	Identity (%)
112	LAREeSSRQ032	JR175164	CCCCCTGCACACCATTTT<br>CAAGAATGCCGATACCGAAT	1.5	56	169	$(TTG)_6$	180	97
113	LAREeSSRQ035	JR175381	CCTCGAACACTCACTAAACTTGC<br>ATGCCTCCTTGTGCATTCTT	1.5	56	112	$(AT)_7$	135	90
114	LAREeSSRQ036	JR175557	TACTTCCCTGTGCTGGGTTT<br>GAAAAAGACTCCCAAAGGGG	1.5	56	175	$(TGC)_6$	181	97
115	LAREeSSRQ048	JR176325	TGAAGAAGAAGCGGAAGAGG<br>AGGCTATACGCTTCCTGCAA	1.5	56	437	$(GAA)_5$	463	97
116	LAREeSSRQ051	JR176852	CGACTCAGCCACCTCGTAAT<br>ATTGCCAGAACCCCTTTTCT	1.5	56	248	$(TA)_8G(TA)_6$	268	96
117	LAREeSSRQ053	JR177135	TGTCGCCTTCACTCTGTGAG<br>ATCAATGCGGTGAAGATTCC	1.5	56	178	$(AT)_6$	182	90
118	LAREeSSRQ066	JR178582	GCTCTTGTTGAGCCACCTTC<br>ATGGTTTGGATGCACATGAA	1.5	56	156	$(CA)_{14}$	182	100
119	LAREeSSRQ067	JR178682	ATCTCCTTGGAATGTGTGCC<br>GGGGCGATTACCCTAAATGT	1.5	56	221	$(TC)_8$	246	90
120	LAREeSSRQ070	JR178932	GCTCCTCTTGCACAGTCTCC<br>TGCTCCATTTGTGGGTGTTA	1.5	56	165	$(TA)_6$	188	95
121	LAREeSSRQ074	JR179414	GTATGAAGAGCACCCCAAGG<br>GCAAATAGTTGCAAGGCATGT	1.5	56	130	$(AT)_8$	164	85
122	LAREeSSRQ104	JR183015	ATCACTGCTCATGAGTCGCA<br>GTATGCGTTTGCGTGTGTGT	1.5	56	208	$(CA)_7$	215	98
123	LAREeSSRQ113	JR184160	TCCAATGGAGGACGTAAAGG<br>TCATGCATCATAACATTGAATAACA	1.5	56	198	$(AC)_{10}$	207	87
124	LAREeSSRQ114	JR185111	GAAACGGATATGGGAATGGA<br>TTGATGAATGGTAATCTGACCTATG	1.5	56	151	$(CA)_7$	161	99
125	LAREeSSRQ115	JR185400	AATTAAATGCGCTCACCTCG<br>GCAGATAACGCAGCCTTCTT	1.5	56	253	$(CTG)_6$	260	90

Table S1. *Cont.*

Serial No.	Genic-SSR Locus	NCBI Accession	Primer Sequence (5'-3')	Mg^{2+} (mM)	Tm (°C)	Target Size (bp)	Repeat Motif in EST	Target Size (Length Resequenced)(bp)	Identity (%)
126	LAREeSSRQ120	JR186302	ATTCCCCATTTCACGAAGC<br>TACTCCGAGAGGAGGCAGAA	1.5	56	127	$(ACTCT)_5$	120	88
127	LAREeSSRQ125	JR186594	AAGGGAAAATAAAGCCCTCG<br>TGCTCTCAGGTTGCAATGAG	1.5	56	125	$(AT)_{10}$	158	90
128	LAREeSSRQ127	JR186781	GGTTTCCATTACAACTCAAGGG<br>GGATTCAGCTTCGCTTTCAC	1.5	56	379	$(ATG)_5$	400	96
129	LAREeSSRQ137	JR188117	GTGCCTTGTGGGTTGTCTTT<br>AAGAGTTGCCACCCATAAGC	1.5	56	276	$(TG)_7$	294	93
130	LAREeSSRQ141	JR188688	CACACATGCAAAGCAAACAA<br>TGTGTGTGAATGTGAGAGGGA	1.5	56	137	$(TC)_9$	157	87
131	LAREeSSRQ183	JR193542	TGTTTGACGGTGACTGAAGG<br>TAGAGGAGCAGCGAGAGGAG	1.5	56	143	$(CTC)_7$	157	85
132	LAREeSSRQ187	JR193964	TGAGGATTCTTTCCCAATGC<br>CATTGGATCCCAAAGGGTAG	1.5	56	177	$(TG)_8$	187	99
133	LAREeSSRQ195	JR194843	GCAGATTGTAGAAGGGCTGC<br>CATCGCCTTTCTCACACAGA	1.5	56	219	$(AGA)_5$	236	85
134	LAREeSSRQ206	JR139531	GCAGACCCATTTTCGTGATT<br>CGCATCTCAGAGGGAGAGAG	1.5	56	448	$(GTT)_5$	460	94
135	LAREeSSRQ209	JR139801	CCACGGAGTTTGGACTGAAT<br>CTAAACAGAGCCCAAGCGTC	1.5	56	185	$(GGA)_5$	197	95
136	LAREeSSRQ210	JR139804	GTTCGATTTTGGCCCATCTA<br>GATCAATTTTGGTTCGTTGTCA	1.5	56	186	$(TA)_9$	196	90
137	LAREeSSRQ213	JR140280	TTTTGCTTTGTGAATGTGGC<br>TGGGATCCTGAGGGACTATG	1.5	56	297	$(TTC)_5$	311	90
138	LAREeSSRQ216	JR140886	ATTTCTGCGGCAAAGAGTTG<br>AGAGAGGAAGGACTTTCGGC	1.5	56	377	$(AT)_8$	360	86
139	LAREeSSRQ218	JR140959	AATTAGTGGTGCTTCGGTGG<br>TGGCACTTCTTGTAAATAAAATCAA	1.5	56	262	$(AT)_6$	270	94

Table S1. *Cont.*

Serial No.	Genic-SSR Locus	NCBI Accession	Primer Sequence (5'-3')	Mg^{2+} (mM)	Tm (°C)	Target Size (bp)	Repeat Motif in EST	Target Size (Length Resequenced)(bp)	Identity (%)
140	LAREeSSRQ235	JR143407	CACCATAAGCAACAGCGAAA<br>GTGCCGATGGATGTCTTTCT	1.5	56	199	$(CAGCAA)_5$	208	99
141	LAREeSSRQ243	JR144253	TTCGTGTACAGCGTTCAAGC<br>TCCGGAATATCGTCACAACA	1.5	56	177	$(AT)_7$	200	94
142	LAREeSSRQ247	JR144913	CTACGAGAGGCTCGATACGC<br>CTTCAGTCTGGAGCTGACCC	1.5	56	459	$(CT)_8$	461	85
143	LAREeSSRQ257	JR146140	TCTGCATCCTAGTGCTGTGG<br>CCCCTGGATCTTCTGAAACA	1.5	56	122	$(ATC)_5$	142	90
144	LAREeSSRQ285	JR149637	CCGAGACATGATGCTGAGAA<br>TATTTGCAGAAGCCCAAACC	1.5	56	171	$(GAG)_5$	196	96
145	LAREeSSRQ299	JR151216	AAACCAATGAAAATGCCTGC<br>TCCCCAGCCAACTCTCATAC	1.5	56	484	$(CTG)_5$	496	91
146	LAREeSSRQ316	JR153273	AGCTCTCTGTGCTTTCTCGC<br>GGAAAGAGCAATTCAGCAGG	1.5	56	205	$(TC)_7$	210	90
147	LAREeSSRQ322	JR153722	AGGCGTCTGAGCTACCAAAA<br>CGACGACACCCAATACCTTT	1.5	56	429	$(CAG)_5$	443	99
148	LAREeSSRQ330	JR154204	CAGGAAGTTGGGCAGCTTAG<br>GGTCTTGGCCTTGTTGTTGT	1.5	56	253	$(TC)_6$	266	94
149	LAREeSSRQ352	JR155690	CCACCTCAAATCTTCTCCCA<br>AGATGGAATACTGTTGGCGG	1.5	56	288	$(GCA)_5$	270	90
150	LAREeSSRQ364	JR157274	GATGAAATTGCGGAAAGCAT<br>ACTGGGCAATGTCCAAACTC	1.5	56	286	$(AT)_7$	315	96
151	LAREeSSRQ375	JR158646	AGTGGCAGTCAGCATCTCCT<br>AGAAGATTTTGCAGAGGGCA	1.5	56	211	$(TC)_6$	235	93
152	LAREeSSRQ377	JR158866	TCATCATCCTCCTCGTCCTC<br>AAGATTCAGTGGATGGCGAC	1.5	56	196	$(TCATCC)_5$tcagtctca gtt$(TCAGTC)_5$	208	90

Table S1. *Cont.*

Serial No.	Genic-SSR Locus	NCBI Accession	Primer Sequence (5'-3')	Mg^{2+} (mM)	Tm (°C)	Target Size (bp)	Repeat Motif in EST	Target Size (Length Resequenced)(bp)	Identity (%)
153	LAREeSSRQ382	JR159113	TGGTTCAACTTCTCTCGCCT	1.5	56	304	$(CAG)_5$	310	82
			GGAATGTGAACGAAGACGGT						
154	LAREeSSRQ386	JR159815	TCCATCTTTATTTGGCAGGC	1.5	56	138	$(GA)_{13}$	155	85
			CCATCAGAGATGGGAGTGCT						
155	LAREeSSRQ393	JR160488	CCTTGTGAAGGGCACAGTTT	1.5	56	372	$(AG)_6$	380	98
			ATGAGGTCTGTGAGGGGTTG						
156	LAREeSSRQ397	JR161052	TCTGAATCAATGTATCATGTATCGAA	1.5	56	137	$(GA)_9$	157	90
			CTGTCAGTCATGCTGCGTTT						
157	LAREeSSRQ399	JR161168	AGACTCCTGTTGGAAAGGCA	1.5	56	254	$(AAG)_5$	271	97
			AGACTCCTGTTGGAAAGGCA						
158	LAREeSSRQ403	JR161642	ACACAACATGCTACGATGCC	1.5	56	225	$(CAT)_8$	255	90
			GCTTCTAGGCGTTCAACGAG						
159	LAREeSSRQ406	JR161926	TGCATTCTGTTAATGCCCAA	1.5	56	384	$(AG)_6$	397	85
			TGTTGATGAGCAATGACCGT						
160	LAREeSSRQ408	JR162009	CAAGCATTCTTCCCCAAAAA	1.5	56	162	$(GACTG)_7$	172	94
			TAAGTCCAGTCCAGTCCGGT						
161	LAREeSSRQ409	JR162187	AAAATTTCATCCTCGAACACTCA	1.5	56	162	$(AT)_9$	172	90
			TGGACAATGTTCCATGCAGT						
162	LAREeSSRQ430	JR166454	TTTTGGTCCGATCAGGAGTC	1.5	56	292	$(CGG)_5(CTG)_2$TTGA $(TGC)_6$tgttgatgctgatg g$(TGC)_8$	312	88
			CAACTTTTGGGTTGGGAGAA						
163	LAREeSSRQ439	JR168298	TCTCGCTCGGCTTCTACATT	1.5	56	255	$(AAT)_5$	264	90
			GAGATTCTGCTGCTTCCCTG						
164	LAREeSSRQ444	JR168664	GAACGTTCAAACTGCACACG	1.5	56	419	$(TGC)_6$	422	99
			TTGAGTTCATTGGCTGCAAG						
165	LAREeSSRQ449	JR169475	CCCTTAGCCTCTTTTTGAGGA	1.5	56	291	$(AT)_8$	300	98
			ACCATCGAACGTGTCAACAA						

Table S2. Polymorphisms and functional annotations of the 165 genic-SSRs.

Serial No.	Genic-SSR Locus	Polymorphisms in *L. kaempferi*					SSR Position	EST Putative at $E \leq 10^{-5}$[Organism]	BlastX *E*-Value
		N_a	ASR	H_e	H_o	PIC			
1	LARKeSSRH002	7	117-156	0.771	0.708	0.722	CDS	No significant match	—
2	LARKeSSRH008*	3	400-414	0.327	0.125	0.294	CDS	No significant match	—
3	LARKeSSRH028	1	198	NC	NC	NC	5'UTR	No significant match	—
4	LARKeSSRH029	1	263	NC	NC	NC	3'UTR	No significant match	—
5	LARKeSSRH034	3	93-111	0.536	0.500	0.424	5'UTR	No significant match	—
6	LARKeSSRH042	2	334-346	0.510	0.458	0.375	3'UTR	transferase, transferring glycosyl groups, putative [Ricinus communis]	2E-18
7	LARKeSSRH045	1	274	NC[a]	NC	NC	CDS	No significant match	—
8	LARKeSSRH046	3	154-174	0.648	0.583	0.557	unknown	No significant match	—
9	LARKeSSRH052	3	333-349	0.377	0.417	0.325	3'UTR	No significant match	—
10	LARKeSSRH057	7	110-130	0.610	0.667	0.553	CDS	No significant match	—
11	LARKeSSRH094	4	253-265	0.478	0.375	0.437	5'UTR	No significant match	—
12	LARKeSSRH106*	2	247-257	0.479	0.417	0.359	5'UTR	NFYA4 [Larix kaempferi]	3E-24
13	LARKeSSRH122*	5	240-264	0.734	0.542	0.675	3'UTR	No significant match	—
14	LARKeSSRH125	6	237-251	0.742	0.458	0.691	CDS	No significant match	—
15	LARKeSSRH128*	2	239-249	0.337	0.250	0.276	3'UTR	No significant match	—
16	LARKeSSRH131	0	287	NC	NC	NC	3'UTR	hypothetical protein POPTR_ 0015s11010g [Populus trichocarpa]	4E-18
17	LARKeSSRH136	2	373-397	0.042	0.042	0.040	CDS	glycosyltransferase, CAZy family GT4 [Selaginella moellendorffii]	2E-09
18	LARKeSSRH137	1	170	NC	NC	NC	5'UTR	No significant match	—
19	LARKeSSRH140	6	294-336	0.559	0.583	0.516	CDS	No significant match	—
20	LARKeSSRH147*	2	181-193	0.042	0.042	0.039	CDS	conserved hypothetical protein [Ricinus communis]	5E-34
21	LARKeSSRH149	3	251-291	0.494	0.458	0.385	CDS	No significant match	—
22	LARKeSSRH168	5	145-169	0.685	0.750	0.617	CDS	hypothetical protein CICLE _v10013564mg [Citrus clementina]	3E-15

Table S2. *Cont.*

Serial No.	Genic-SSR Locus	Polymorphisms in *L. kaempferi*					SSR Position	EST Putative at $E \leq 10^{-5}$[Organism]	BlastX *E*-Value
		N_a	ASR	H_e	H_o	PIC			
23	LARKeSSRH177	3	394-414	0.513	0.500	0.396	CDS	No significant match	—
24	LARKeSSRH179	1	335	NC	NC	NC	CDS	No significant match	—
25	LARKeSSRH180	2	169-178	0.361	0.375	0.291	CDS	No significant match	—
26	LARKeSSRH182	1	314	NC	NC	NC	CDS	No significant match	—
27	LARKeSSRH187	3	123-141	0.407	0.500	0.354	CDS	SCL5 [Pinus radiata]	2E-07
28	LARKeSSRH189	4	255-273	0.480	0.500	0.393	CDS	predicted protein [Physcomitrella patens]	8E-04
29	LARKeSSRH191	3	145-163	0.507	0.583	0.408	5'UTR	No significant match	—
30	LARKeSSRH197	2	327-337	0.337	0.333	0.276	3'UTR	GID1-like gibberellin receptor [Larix kaempferi]	2E-72
31	LARKeSSRH206	3	312-362	0.414	0.375	0.369	5'UTR	No significant match	—
32	LAREeSSRH217	3	418-436	0.643	0.708	0.553	CDS	ethylene response factor ERF2 [Eriobotrya japonica]	5E-13
33	LARKeSSRH221*	4	376-394	0.688	0.708	0.609	CDS	hypothetical protein JCGZ_21189 [Jatropha curcas]	5E-176
34	LARKeSSRH224	2	177-184	0.082	0	0.077	5'UTR	No significant match	—
35	LARKeSSRH233	3	444-456	0.566	0.625	0.493	CDS	hypothetical protein EUTSA_ v10018329mg [Eutrema salsugineum]	3E-134
36	LARKeSSRH236	6	300-321	0.505	0.458	0.463	5'UTR	No significant match	—
37	LARKeSSRH239	2	333-342	0.403	0.542	0.317	3'UTR	No significant match	—
38	LARKeSSRH251	3	125-143	0.407	0.500	0.354	CDS	SCL5 [Pinus radiata]	2E-08
39	LARKeSSRH253	2	342-366	0.042	0.042	0.039	CDS	TCP family transcription factor, putative [Theobroma cacao]	1E-51
40	LARKeSSRH256	3	113-125	0.659	0.875	0.571	5'UTR	Cysteine-rich receptor-like protein kinase 25 [Aegilops tauschii]	1E-20
41	LARKeSSRH264	6	96-132	0.691	0.667	0.637	CDS	No significant match	—
42	LARKeSSRH274	5	252-272	0.509	0.375	0.447	unknown	No significant match	—

Table S2. *Cont.*

Serial No.	Genic-SSR Locus	Polymorphisms in *L. kaempferi*					SSR Position	EST Putative at $E \leq 10^{-5}$[Organism]	BlastX *E*-Value
		N_a	ASR	H_e	H_o	PIC			
43	LARKeSSRH276	5	154-189	0.650	1	0.575	CDS	No significant match	—
44	LARKeSSRH279*	4	160-187	0.734	0.583	0.668	5'UTR	No significant match	—
45	LARKeSSRH283*	4	331-367	0.233	0.083	0.219	3'UTR	No significant match	—
46	LARKeSSRH299	5	149-173	0.685	0.750	0.617	CDS	hypothetical protein AMTR_ s00060p00187780 [Amborella trichopoda]	1E-44
47	LARKeSSRH301	4	256-280	0.439	0.417	0.388	CDS	hypothetical protein M569_06112 [Genlisea aurea]	7E-05
48	LARKeSSRH309	1	466	NC	NC	NC	CDS	protein LYK5-like [Nelumbo nucifera]	4E-28
49	LARKeSSRH339	2	316-319	0.190	0.208	0.169	3'UTR	PREDICTED: zinc finger protein 8-like [Musa acuminata subsp. malaccensis]	5E-06
50	LAREeSSRHL003	5	304-324	0.691	0.833	0.618	5'UTR	reverse transcriptase [Ginkgo biloba]	6E-16
51	LAREeSSRHL004	5	200-224	0.709	0.541	0.642	CDS	No significant match	—
52	LAREeSSRHL006*	4	99-117	0.577	0.708	0.489	CDS	No significant match	—
53	LAREeSSRHL007	4	238-278	0.267	0.125	0.248	CDS	No significant match	—
54	LAREeSSRHL009	6	151-175	0.768	0.958	0.713	CDS	No significant match	—
55	LAREeSSRHL021	2	160-170	0.082	0.083	0.077	CDS	No significant match	—
56	LAREeSSRHL034	8	92-116	0.717	0.708	0.658	CDS	hypothetical protein 0_3046_01 [Pinus mugo]	1E-16
57	LAREeSSRHL042*	6	87-117	0.759	0.583	0.700	CDS	No significant match	—
58	LAREeSSRHL046	1	380	NC	NC	NC	CDS	No significant match	—
59	LAREeSSRHL062*	3	222-242	0.513	0.458	0.396	3'UTR	No significant match	—
60	LAREeSSRHL079	5	172-202	0.645	0.583	0.576	3'UTR	multicopper oxidase LPR2 [Solanum lycopersicum]	1E-12
61	LAREeSSRHL083	1	142	NC	NC	NC	CDS	No significant match	—
62	LAREeSSRHL085*	4	123-141	0.197	0.208	0.185	CDS	No significant match	—
63	LAREeSSRHL101*	4	232-250	0.657	0.125	0.593	CDS	class I chitinase [Pinus contorta]	2E-51
64	LAREeSSRHL104*	7	283-313	0.791	0.542	0.740	CDS	No significant match	—

Table S2. *Cont.*

Serial No.	Genic-SSR Locus	Polymorphisms in *L. kaempferi*					SSR Position	EST Putative at $E \leq 10^{-5}$[Organism]	BlastX *E*-Value
		N_a	ASR	H_e	H_o	PIC			
65	LAREeSSRHL114	7	187-232	0.761	0.750	0.708	CDS	No significant match	—
66	LAREeSSRHL120	3	133-145	0.481	0.500	0.378	3'UTR	No significant match	—
67	LAREeSSRHL129	0	254	NC	NC	NC	3'UTR	No significant match	—
68	LAREeSSRHL137	4	100-112	0.605	0.708	0.545	CDS	No significant match	—
69	LAREeSSRHL138	2	243-252	0.496	0.500	0.368	CDS	No significant match	—
70	LAREeSSRHL159	3	224-236	0.435	0.541	0.380	CDS	No significant match	—
71	LAREeSSRHL161	6	302-332	0.570	0.500	0.517	CDS	No significant match	—
72	LAREeSSRHL162*	9	85-119	0.814	0.792	0.772	unknown	No significant match	—
73	LAREeSSRHL163*	4	263-287	0.659	0.458	0.596	CDS	No significant match	—
74	LAREeSSRHL165	1	382	NC	NC	NC	CDS	No significant match	—
75	LAREeSSRHL166	1	207	NC	NC	NC	3'UTR	No significant match	—
76	LAREeSSRHL215*	2	203-209	0.478	0.500	0.359	CDS	No significant match	—
77	LAREeSSRHL217*	3	204-249	0.519	0.291	0.415	3'UTR	No significant match\	—
78	LAREeSSRHL246	5	188-218	0.398	0.208	0.371	3'UTR	No significant match	—
79	LAREeSSRHL272	3	354-372	0.165	0.174	0.154	unknown	No significant match	—
80	LAREeSSRHL275*	2	354-362	0.467	0.292	0.353	5'UTR	No significant match	—
81	LAREeSSRHL283	2	188-198	0.481	0.500	0.378	unknown	Spore coat protein A [Morus notabilis]	5E-10
82	LAREeSSRHL299	2	446-456	0.403	0.375	0.317	3'UTR	No significant match	—
83	LAREeSSRHL308	1	286	NC	NC	NC	unknown	No significant match	—
84	LAREeSSRHL346	3	99-111	0.260	0.292	0.231	CDS	No significant match	—
85	LAREeSSRHL357	2	162-168	0.496	0.500	0.368	CDS	No significant match	—
86	LAREeSSRHL358	2	135-145	0.120	0.125	0.110	CDS	No significant match	—
87	LAREeSSRHL361*	3	275-311	0.121	0.125	0.115	CDS	No significant match	—
88	LAREeSSRHL366*	3	244-256	0.571	0.333	0.471	unknown	No significant match	—
89	LAREeSSRHL372	3	114-128	0.574	0.417	0.482	CDS	No significant match	—
90	LAREeSSRHL374	9	246-268	0.852	0.792	0.816	CDS	hypothetical protein MTR_5g014270 [Medicago truncatula]	4E-04

Table S2. *Cont.*

Serial No.	Genic-SSR Locus	Polymorphisms in *L. kaempferi*					SSR Position	EST Putative at $E \leq 10^{-5}$[Organism]	BlastX *E*-Value
		N_a	ASR	H_e	H_o	PIC			
91	LAREeSSRHL380	3	184-202	0.478	0.542	0.410	CDS	R2R3-MYB transcription factor MYB4 [Picea glauca]	4E-33
92	LAREeSSRHL391	2	224-230	0.191	0.125	0.169	CDS	No significant match	—
93	LAREeSSRHL392*	4	306-324	0.557	0.458	0.466	CDS	hypothetical protein AMTR _s00067p00052610 [Amborella trichopoda]	5E-16
94	LAREeSSRHL393	3	296-302	0.542	0.542	0.453	3'UTR	TATA-binding protein-associated factor 2N-like isoform X1 [Elaeis guineensis]	1E-39
95	LAREeSSRHL394*	2	255-261	0.191	0.208	0.169	CDS	No significant match	—
96	LAREeSSRHL395*	6	272-308	0.699	0.458	0.641	CDS	hypothetical protein 0_16146_01 [Pinus taeda]	4E-46
97	LAREeSSRHL396	5	308-332	0.590	0.500	0.533	CDS	No significant match	—
98	LAREeSSRHL397*	6	223-273	0.713	0.542	0.645	CDS	No significant match	—
99	LAREeSSRHL398*	2	293-299	0.120	0.125	0.110	CDS	predicted protein [physcomitrella patena]	2E-77
100	LAREeSSRHL399	4	275-305	0.357	0.250	0.320	CDS	TB510 [Taxus baccata]	3E-08
101	LAREeSSRHL400	4	272-326	0.565	0.875	0.457	CDS	No significant match	
102	LAREeSSRHL401	2	302-320	0.254	0.292	0.218	CDS	hypothetical protein POPTR _0010s02550g [Populus trichocarpa]	1E-09
103	LAREeSSRHL402	9	242-270	0.814	0.833	0.771	CDS	No significant match	—
104	LAREeSSRHL403*	3	286-290	0.518	0.125	0.428	CDS	No significant match	—
105	LARKeSSRHL404	1	299	NC	NC	NC	CDS	No significant match	—
106	LAREeSSRQ001	6	182-210	0.504	0.333	0.460	CDS	No significant match	—
107	LAREeSSRQ005	4	246-252	0.634	0.750	0.546	CDS	No significant match	—
108	LAREeSSRQ006	6	131-175	0.512	0.542	0.475	CDS	No significant match	—
109	LAREeSSRQ010	2	216, 222	NC	NC	NC	CDS	No significant match	—
110	LAREeSSRQ017*	5	127-139	0.714	0.625	0.647	unknown	No significant match	—
111	LAREeSSRQ020	3	219-231	0.230	0.083	0.212	3'UTR	No significant match	—

Table S2. *Cont.*

Serial No.	Genic-SSR Locus	Polymorphisms in *L. kaempferi*					SSR Position	EST Putative at $E \leq 10^{-5}$[Organism]	BlastX *E*-Value
		N_a	ASR	H_e	H_o	PIC			
112	LAREeSSRQ032	2	152-170	0.488	0.542	0.364	5'UTR	No significant match	—
113	LAREeSSRQ035	5	108-118	0.740	0.833	0.676	3'UTR	No significant match	—
114	LAREeSSRQ036*	6	207-219	0.636	0.542	0.565	CDS	low temperature and salt responsive protein [Medicago sativa]	5E-23
115	LAREeSSRQ048*	2	434-461	0.042	0.042	0.039	CDS	No significant match	—
116	LAREeSSRQ051	13	234-268	0.870	0.542	0.838	5'UTR	No significant match	—
117	LAREeSSRQ053	6	167-181	0.683	1	0.613	CDS	hypothetical protein glysoja_008847 [Glycine soja]	2E-08
118	LAREeSSRQ066	3	142-156	0.680	0.708	0.592	CDS	No significant match	—
119	LAREeSSRQ067	6	221-233	0.483	0.500	0.448	CDS	No significant match	—
120	LAREeSSRQ070*	12	164-198	0.900	0.792	0.870	unknown	No significant match	—
121	LAREeSSRQ074*	11	124-146	0.867	0.542	0.834	unknown	No significant match	—
122	LAREeSSRQ104	6	205-233	0.629	0.750	0.547	CDS	No significant match	—
123	LAREeSSRQ113	8	184-204	0.822	0.875	0.778	5'UTR	No significant match	—
124	LAREeSSRQ114*	6	129-147	0.537	0.458	0.499	3'UTR	No significant match	—
125	LAREeSSRQ115	3	317-332	0.513	0.542	0.396	CDS	No significant match	—
126	LAREeSSRQ120*	2	110-115	0.383	0.416	0.305	3'UTR	No significant match	—
127	LAREeSSRQ125	11	100-144	0.864	0.458	0.830	CDS	No significant match	—
128	LAREeSSRQ127	3	371-377	0.630	1	0.544	5'UTR	No significant match	—
129	LAREeSSRQ137	3	272-278	0.122	0.125	0.115	CDS	No significant match	—
130	LAREeSSRQ141	5	133-141	0.302	0.333	0.282	CDS	No significant match	—
131	LAREeSSRQ183	4	126-141	0.424	0.500	0.358	CDS	HAT transposon superfamily isoform 4 [Theobroma cacao]	5E-06
132	LAREeSSRQ187*	5	178-190	0.423	0.458	0.387	CDS	No significant match	—
133	LAREeSSRQ195	2	220-223	0.191	0.042	0.169	CDS	No significant match	—
134	LAREeSSRQ206	5	446-470	0.630	0.583	0.548	CDS	No significant match	—

Table S2. *Cont.*

Serial No.	Genic-SSR Locus	Polymorphisms in *L. kaempferi*					SSR Position	EST Putative at $E \leq 10^{-5}$[Organism]	BlastX *E*-Value
		N_a	ASR	H_e	H_o	PIC			
135	LAREeSSRQ209	3	182-188	0.465	0.583	0.410	5'UTR	glucan endo-1,3-beta-glucosidase 13 isoform X1 [Phoenix dactylifera]	2E-44
136	LAREeSSRQ210	8	165-191	0.700	0.792	0.660	5'UTR	No significant match	—
137	LAREeSSRQ213	1	300	NC	NC	NC	3'UTR	No significant match	—
138	LAREeSSRQ216	3	374-406	0.263	0.292	0.239	3'UTR	No significant match	—
139	LAREeSSRQ218*	4	245-281	0.357	0.208	0.320	3'UTR	No significant match	—
140	LAREeSSRQ235*	3	183-195	0.411	0.500	0.363	CDS	No significant match	—
141	LAREeSSRQ243*	13	149-185	0.903	0.583	0.874	5'UTR	hypothetical protein PRUPE _ppa014495mg [Prunus persica]	6E-14
142	LAREeSSRQ247	8	428-466	0.546	0.583	0.516	5'UTR	No significant match	—
143	LAREeSSRQ257	4	116-131	0.574	0.667	0.475	CDS	No significant match	—
144	LAREeSSRQ285	7	162-192	0.710	0.792	0.658	CDS	putative wall-associated protein kinase [Pinus massoniana]	3E-09
145	LAREeSSRQ299	2	431-485	0.283	0.333	0.239	CDS	R2R3-MYB transcription factor MYB9 [Picea glauca]	7E-15
146	LAREeSSRQ316	2	194-206	0.311	0.292	0.258	CDS	No significant match	—
147	LAREeSSRQ322*	3	426-459	0.443	0.458	0.369	CDS	hypothetical protein JCGZ_07669 [Jatropha curcas]	8E-120
148	LAREeSSRQ330*	3	255-267	0.529	0.042	0.403	CDS	hypothetical protein CICLE_ v10001970mg [Citrus clementina]	6E-67
149	LAREeSSRQ352*	5	249-288	0.738	0.792	0.674	CDS	WRKY family transcription factor [Theobroma cacao]	3E-81
150	LAREeSSRQ364*	11	280-306	0.883	0.542	0.851	5'UTR	No significant match	—
151	LAREeSSRQ375	3	212-230	0.465	0.500	0.410	3'UTR	No significant match	—
152	LAREeSSRQ377*	6	178-208	0.665	0.417	0.589	5'UTR	No significant match	—
153	LAREeSSRQ382	1	299	NC	NC	NC	CDS	mys transcription factor [Pinus sylvestris]	2E-40

Table S2. *Cont.*

Serial No.	Genic-SSR Locus	Polymorphisms in *L. kaempferi*					SSR Position	EST Putative at $E \leq 10^{-5}$[Organism]	BlastX *E*-Value
		N_a	ASR	H_e	H_o	PIC			
154	LAREeSSRQ386	9	128-148	0.818	0.833	0.779	CDS	No significant match	—
155	LAREeSSRQ393	1	371	NC	NC	NC	5'UTR	No significant match	—
156	LAREeSSRQ397	10	132-154	0.829	0.792	0.789	CDS	No significant match	—
157	LAREeSSRQ399*	2	254-263	0.467	0.458	0.353	5'UTR	hypothetical protein CL1530Contig1 _04 [Pinus radiata]	3E-27
158	LAREeSSRQ403	9	216-246	0.765	0.708	0.710	5'UTR	No significant match	—
159	LAREeSSRQ406	4	361-385	0.389	0.375	0.352	unknown	No significant match	—
160	LAREeSSRQ408	6	144-180	0.693	0.833	0.620	CDS	No significant match	—
161	LAREeSSRQ409	8	181-199	0.786	0.875	0.736	CDS	No significant match	—
162	LAREeSSRQ430	9	286-313	0.811	0.875	0.764	CDS	sequence-specific DNA binding transcription factor [Arabidopsis thaliana]	4E-39
163	LAREeSSRQ439	1	258	NC	NC	NC	5'UTR	hypothetical protein JCGZ_04489 [Jatropha curcas]	2E-84
164	LAREeSSRQ444*	2	406-415	0.156	0.167	0.141	CDS	No significant match	—
165	LAREeSSRQ449*	5	272-298	0.552	0.208	0.486	5'UTR	No significant match	—
	[b] Mean	4.510		0.518	0.487	0.459			

[a] NC = not calculated (20 genic-SSRs) due to monomorphism among 24 genotypes; [b] Mean value over the 145 polymorphic genic-SSRmarkers; * indicates significant deviation from Hardy-Weiberg equilibrium ($p < 0.001$).

Table S3. Amplification rate (AMAR) of 30 *Larix kaempferi* genic-SSR markers in other *Larix* species.

Primer Name	Species		
	Larix olgensis	*Larix principi-rupprechtii*	*larix gmelinii*
LARKeSSRH042	+	+	+
LARKeSSRH046	+	+	+
LARKeSSRH106	+	+	+
LARKeSSRH149	+	−	+
LARKeSSRH191	+	+	+
LARKeSSRH224	−	+	−
LARKeSSRH264	+	+	+
LARKeSSRH299	+	−	−
LAREeSSRHL004	+	+	+
LAREeSSRHL034	+	+	+
LAREeSSRHL062	+	+	+
LAREeSSRHL101	−	+	−
LAREeSSRHL138	+	+	+
LAREeSSRHL162	−	−	+
LAREeSSRHL246	+	+	+
LAREeSSRHL358	+	+	+
LAREeSSRHL374	−	+	−
LAREeSSRHL392	+	+	+
LAREeSSRHL398	+	+	+
LAREeSSRHL399	+	+	+
LAREeSSRQ005	+	+	+
LAREeSSRQ020	−	+	+
LAREeSSRQ115	+	+	+
LAREeSSRQ125	+	+	+
LAREeSSRQ183	+	+	+
LAREeSSRQ209	+	+	+
LAREeSSRQ243	−	+	−
LAREeSSRQ316	+	+	+
LAREeSSRQ377	+	−	+
LAREeSSRQ449	+	+	+
AMAR	80.0%	86.7%	83.0%

+ = present of PVR amplicons; − = absent of PCR amplicons.
